# Effect of Growth Stage on the Efficacy of Postemergence Herbicides on Four Weed Species of Direct-Seeded Rice

**DOI:** 10.1100/2012/123071

**Published:** 2012-04-24

**Authors:** Bhagirath Singh Chauhan, Seth Bernard Abugho

**Affiliations:** Crop and Environmental Sciences Division, International Rice Research Institute, Los Baños, Philippines

## Abstract

The efficacy of bispyribac-sodium, fenoxaprop + ethoxysulfuron, and penoxsulam + cyhalofop was evaluated against barnyardgrass, Chinese sprangletop, junglerice, and southern crabgrass when applied at four-, six-, and eight-leaf stages. When applied at the four-leaf stage, bispyribac-sodium provided greater than 97% control of barnyardgrass, junglerice, and southern crabgrass; however, it was slightly weak (74% control) on Chinese sprangletop. Irrespective of the weed species, fenoxaprop + ethoxysulfuron provided greater than 97% control when applied at the four-leaf stage. At the same leaf stage, penoxsulam + cyhalofop controlled 89 to 100% barnyardgrass, Chinese sprangletop, and junglerice and only 54% of southern crabgrass. The efficacy of herbicides was reduced when applied at the eight-leaf stage of the weeds; however, at this stage, fenoxaprop + ethoxysulfuron was effective in controlling 99% of Chinese sprangletop. The results demonstrate the importance of early herbicide application in controlling the weeds. The study identified that at the six-leaf stage of the weeds, fenoxaprop + ethoxysulfuron can effectively control Chinese sprangletop and southern crabgrass, penoxsulam + cyhalofop can effectively control Chinese sprangletop, and bispyribac-sodium can effectively control junglerice.

## 1. Introduction

Rice is an important crop in Asia, where 90% of this crop is grown and consumed. Rice is traditionally grown by transplanting seedlings into puddled soil. Recently, due to high labor cost and less availability of water, there has been a trend to shift from transplanting to direct-seeded rice (DSR) in many Asian countries [1]. However, weeds are a greater problem in DSR than in transplanted rice because of the absence of the crop seedling size advantage and standing water at the time of crop emergence [2].

Many weeds, including barnyardgrass (*Echinochloa crus-galli* (L.) Beauv.), Chinese sprangletop (*Leptochloa chinensis *L.), junglerice (*Echinochloa colona *(L.) Link), and southern crabgrass (*Digitaria ciliaris* (Retz.) Koel.) are problematic grass species in DSR [3, 4]. Barnyardgrass and junglerice are examples of “crop mimicry” as they closely resemble rice at the seedling stage. By the time these weeds can be easily recognized by farmers, crop yield losses may already be inevitable [5]. In DSR, junglerice and southern crabgrass seedling emergence was greater in a no-till system compared with a conventionally tilled system [6]. In another study, rice residue of up to 4 Mg ha^−1^ was not able to reduce the growth of barnyardgrass [7], suggesting that the crop residue at this amount, as a mulch on the soil surface, may not provide suppression of this weed. Barnyardgrass at a density of 9 plants m^−2^ can reduce rice yield by more than 50% [8] and heavy infestation of this weed can remove up to 80% of the nitrogen (N) from the soil [9]. In a recent study, Chinese sprangletop grown under rice interference responded with increased leaf area ratio (amount of leaf area per unit plant dry biomass) and specific leaf area (amount of leaf area per unit leaf biomass), reflecting changes in the distribution of leaf biomass under competition [10]. Furthermore, Chinese sprangletop was not a prevalent and dominant weed in rice fields of Malaysia while transplanting of rice was the usual establishment method, but it became widespread with the shift to DSR [11]. These studies suggest the importance of these weed species in DSR.

Postemergence herbicides are a major tool used to control weeds in DSR. The growth stage of weed species may have an effect on herbicide efficacy by influencing uptake and metabolism of herbicides [12]. Diclofop, for example, was more effective on green foxtail (*Setaria viridis* (L.) Beauv.) and wild oat (*Avena fatua* L.) when applied at an early growth stage [13]. Conversely, trifloxysulfuron was more effective on yellow nutsedge (*Cyperus esculentus* L.) at late application stages [12]. Generally, the herbicide efficacy is lower when applied on bigger weeds. The herbicide degradation rate may be faster in big plants, and herbicide rates may need to be increased to achieve the same level of control [12]. In addition, reliance on a single herbicide may result in evolution of herbicide resistance in weeds and shift in weed flora. In Sri Lanka, for example, continuous use of bispyribac-sodium to control propanil-resistant barnyardgrass has resulted in a shift to dominance by Chinese sprangletop in rice [14]. There are reports from India that Chinese sprangletop is poorly controlled by bispyribac-sodium [15]. Therefore, optimum time of herbicide application and range of herbicides may help control these weeds effectively.

The objective of this study was to evaluate the efficacy of different postemergence herbicides on barnyardgrass, Chinese sprangletop, junglerice, and southern crabgrass when applied at their different growth stages.

## 2. Materials and Methods

### 2.1. Experimental Details

Greenhouse experiments were conducted at the International Rice Research Institute, Los Baños, Philippines. Twenty-five seeds, each of barnyardgrass, Chinese sprangletop, junglerice, and southern crabgrass were planted on the soil surface in plastic trays (8 cm by 8 cm by 5 cm). Soil used in this study was collected from upland rice fields and it had a pH of 6.6 with 31% sand, 37% silt, and 32% clay. Seedlings were thinned to 10 plants per tray immediately after emergence. No fertilizer was applied to the weeds.

Plants were sprayed at the four-, six-, and eight-leaf stages using a research track sprayer that delivered 210 L ha^−1^ spray solution at a spray pressure of 140 kPa. Flat nozzles (Teejet 80015) were used in the sprayer. The postemergence herbicide treatments included bispyribac-sodium at 30 g ai ha^−1^ and commercial mixtures of fenoxaprop-p-ethyl + ethoxysulfuron at 45 g ai ha^−1^, and penoxsulam + cyhalofop at 72 g ai ha^−1^. There were control treatments for each leaf stage and weed species in which herbicides were not sprayed.

Plants were watered daily such that there was no water stress in the plants. Seedling survival was determined 14 d after herbicide application with the criterion of at least one green leaf on the plant. Aboveground biomass was measured after drying plant samples in an oven at 70°C for 72 h and expressed as percent control.

### 2.2. Statistical Analyses

The experiments with each weed species were conducted twice using a randomized complete block design with five replicates. Because there was no significant interaction between treatments and experiments, the data from the repeated experiments were pooled (*n* = 10) before being subjected to ANOVA (GenStat 8.0 2005). The data were analyzed separately for each leaf stage by using one-way ANOVA. Mean comparisons were performed based on least significant difference test at 0.05 probability.

## 3. Results and Discussion

### 3.1. Barnyardgrass

**Figure 1:**
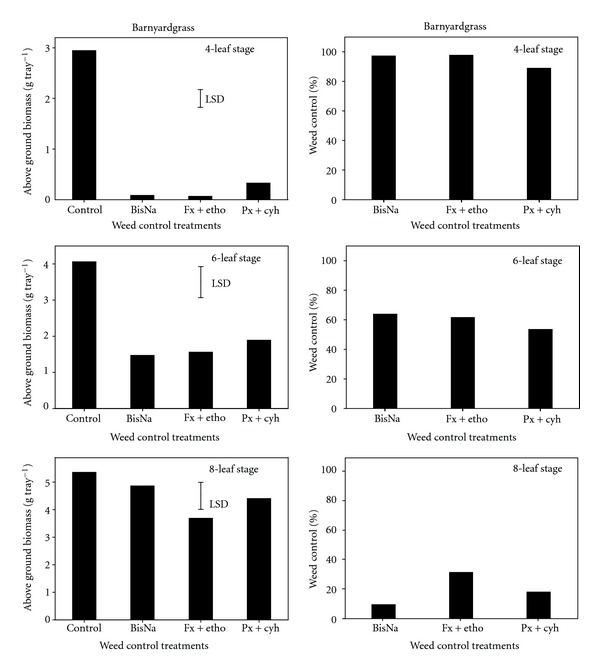
Effect of postemergence herbicides (BisNa, bispyribac-sodium; Fx + etho, fenoxaprop-p-ethyl + ethoxysulfuron; Px + cyh, penoxsulam + cyhalofop) on aboveground dry biomass (g) and control (%) of barnyardgrass when sprayed at its four-, six-, and eight-leaf stages. Mean comparisons were performed based on least significant difference test at 5%.

All herbicides had a phytotoxic effect on plant survival when applied at the early growth stage of barnyardgrass. Delayed herbicide application after the four-leaf stage increased the number of surviving plants, and all plants of barnyardgrass survived when bispyribac-sodium and penoxsulam + cyhalofop were applied at its eight-leaf stage (Table 1). Irrespective of the growth stage of barnyardgrass, there was no difference in efficacy of these herbicides and the biomass and percent weed control decreased with the progress in weed growth (Figure 1). The application of all herbicides gave 89–98% weed control of barnyardgrass when applied at the four-leaf stage. However, delayed application from the four-leaf to the six-leaf stages reduced the control to 53–64%. Similarly, in a previous study in India, fenoxaprop + ethoxysulfuron (150 + 18 g ai ha^−1^) sprayed 21 d after sowing in dry-seeded rice gave 68% control of barnyardgrass [16]. In our study, very poor weed control (9–31%) of barnyardgrass (3.7–4.9 g biomass tray^−1^ in herbicide treatments versus 5.4 g biomass tray^−1^ in the untreated control) was achieved when these herbicides were applied at their eight-leaf stage.

### 3.2. Chinese Sprangletop

No (or very few) seedlings of Chinese sprangletop survived when fenoxaprop + ethoxysulfuron and penoxsulam + cyhalofop were applied at its four- and six-leaf stages (Table 1). However, when penoxsulam + cyhalofop were applied at the eight-leaf stage, more than 60% Chinese sprangletop seedlings survived (with at least one green leaf). Chinese sprangletop sprayed with bispyribac-sodium had more surviving plants than those sprayed with the other two herbicides, and this was true at all leaf stages. Bispyribac-sodium at any stage of Chinese sprangletop was not able to kill more than 10% seedlings (Table 1).

**Figure 2:**
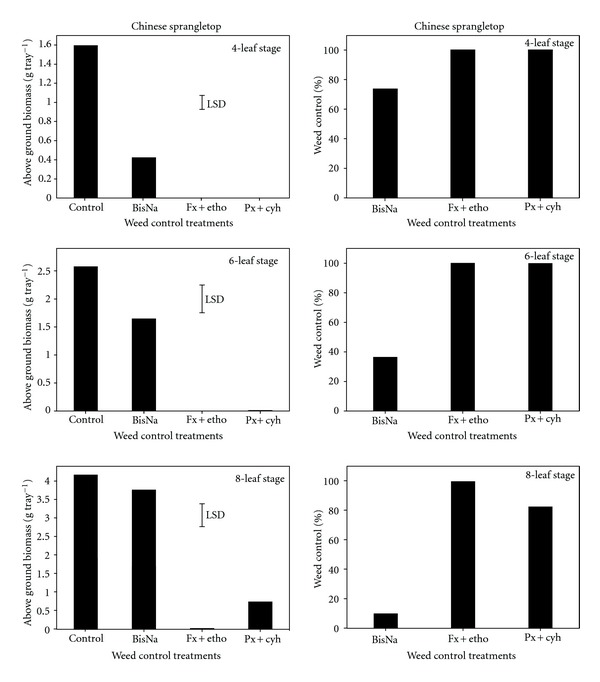
Effect of postemergence herbicides (BisNa, bispyribac-sodium; Fx + etho, fenoxaprop-p-ethyl + ethoxysulfuron; Px + cyh, penoxsulam + cyhalofop) on aboveground dry biomass (g) and control (%) of Chinese sprangletop when sprayed at its four-, six-, and eight-leaf stages. Mean comparisons were performed based on least significant difference test at 5%.

Penoxsulam + cyhalofop gave more than 99% weed control when applied at the four-to-six-leaf stage (Figure 2). The control of Chinese sprangletop with this herbicide was slightly reduced (82% control) when application was delayed to the eight-leaf stage. Irrespective of leaf stage, fenoxaprop + ethoxysulfuron was very effective in controlling (>99%) Chinese sprangletop (Figure 2). On the other hand, the effect of bispyribac-sodium on this weed was poor and control decreased with progress in weed growth (74, 36, and 10% control at the four-, six-, and eight-leaf stages, resp.). This is consistent with reports from India where bispyribac-sodium was shown to be weak on Chinese sprangletop [15]. Penoxsulam alone was also not effective in controlling Chinese sprangletop [15]; however, its commercial mixture with cyhalofop was able to control this weed effectively.

### 3.3. Junglerice

**Figure 3:**
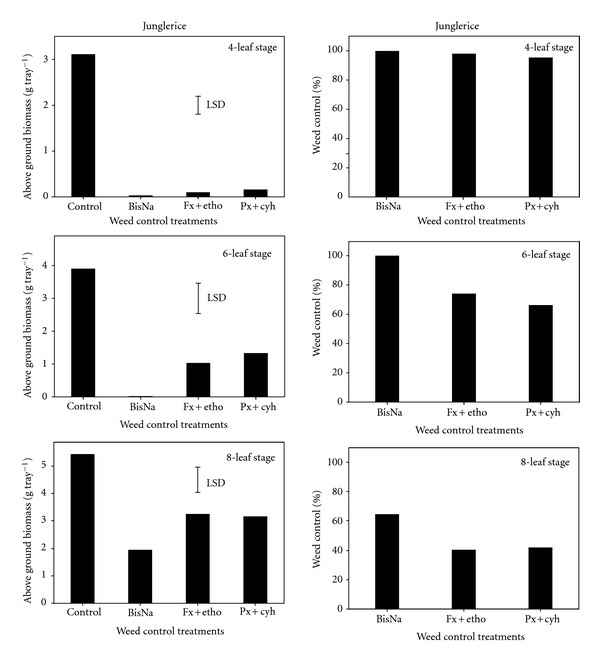
Effect of postemergence herbicides (BisNa, bispyribac-sodium; Fx + etho, fenoxaprop-p-ethyl + ethoxysulfuron; Px + cyh, penoxsulam + cyhalofop) on aboveground dry biomass (g) and control (%) of junglerice when sprayed at its four-, six-, and eight-leaf stages. Mean comparisons were performed based on least significant difference test at 5%.

As observed in barnyardgrass, all herbicides had a phytotoxic effect on junglerice survival when applied at the early stage; however, delayed herbicide application increased the number of surviving plants (Table 1). As compared with the untreated control, the herbicides used in our study reduced more than 95% junglerice biomass when applied at its four-leaf stage (Figure 3). Delayed application of fenoxaprop + ethoxysulfuron and penoxsulam + cyhalofop from the four-to-six-leaf stage reduced junglerice control to 66–74%; however, bispyribac-sodium still gave greater than 99% junglerice control at this stage. Recently, this herbicide has been widely used by farmers in India and Sri Lanka to control junglerice. Weed control by all herbicides was reduced to 40–64% when applied at the eight-leaf stage. Earlier, fenoxaprop + ethoxysulfuron (150 + 18 g ai ha^−1^) sprayed 21 d after rice sowing gave 83% control of junglerice; however, the study did not mention the leaf stage of the weed [16].

### 3.4. Southern Crabgrass

**Figure 4:**
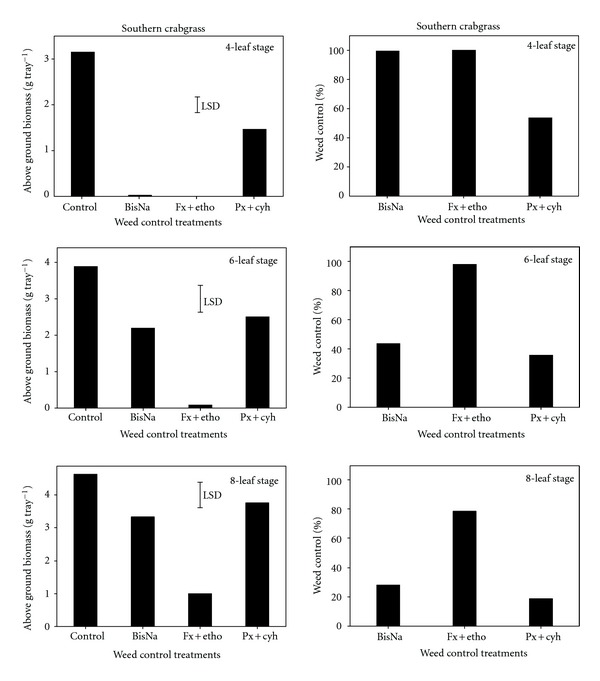
Effect of postemergence herbicides (BisNa, bispyribac-sodium; Fx+etho, fenoxaprop-p-ethyl + ethoxysulfuron; Px+cyh, penoxsulam + cyhalofop) on aboveground dry biomass (g) and control (%) of southern crabgrass when sprayed at its four-, six-, and eight-leaf stages. Mean comparisons were performed based on least significant difference test at 5%.

Only a few plants (<10%) of southern crabgrass survived when fenoxaprop + ethoxysulfuron were applied at the four- or six-leaf stages and greater than 90% plants survived when penoxsulam + cyhalofop were applied at these same stages (Table 1). Bispyribac and fenoxaprop + ethoxysulfuron gave more than 99% control of southern crabgrass when applied at the four-leaf stage (Figure 4). However, the efficacy of bispyribac was reduced markedly when application was delayed from four-to-six-leaf stage. Fenoxaprop + ethoxysulfuron was the most effective herbicide in controlling southern crabgrass at the six- (98%) and eight-leaf stage (78%). Penoxsulam + cyhalofop, on the other hand, did not effectively control southern crabgrass at any leaf stage: 54, 36, and 19% control at the four-, six-, and eight-leaf stage, respectively (Figure 4). This could be due to lower herbicide uptake translocation or to faster metabolism [12].

## 4. Conclusions

The results demonstrate the importance of early herbicide application in controlling the weeds. Generally, the effectiveness of herbicides was low on weeds when applied at their eight-leaf stage. The herbicide degradation rate or metabolism could be faster in big plants, thus herbicide rates may need to be increased to achieve the same level of control [12]. In situations such as rains, farmers may not be able to apply postemergence herbicides at the early stage. Our study identified that at the six-leaf stage of the weeds, fenoxaprop + ethoxysulfuron can effectively control Chinese sprangletop and southern crabgrass, penoxsulam + cyhalofop can effectively control Chinese sprangletop, and bispyribac-sodium can effectively control junglerice. It has been suggested that the control of barnyardgrass is improved with the addition of urea ammonium nitrate to bispyribac [17]. However, it is also possible that the interaction between herbicide and fertilizer may influence the amount of crop injury [3]. Fenoxaprop may cause injury on rice plants. Further research is therefore needed to understand the interaction of these postherbicides and nitrogen fertilizer on rice growth and yield.

## Figures and Tables

**Table 1:** Effect of postemergence herbicides on seedling survival (%) of barnyardgrass, Chinese sprangletop, junglerice, and southern crabgrass when sprayed at their four-, six-, and eight-leaf stage.

Weed control treatment	Rate	Seedling survival		
		Four-leaf	Six-leaf	Eight-leaf
	g ha^−1^		%	
Barnyardgrass				
Untreated control	0	100	100	100
Bispyribac-sodium	30	19	94	100
Fenoxaprop-p-ethyl + ethoxysulfuron	45	13	56	80
Penoxsulam + cyhalofop	72	63	83	100
LSD_0.05_		17	22	12

Chinese sprangletop				
Untreated control	0	100	100	100
Bispyribac-sodium	30	91	99	100
Fenoxaprop-p-ethyl + ethoxysulfuron	45	0	1	1
Penoxsulam + cyhalofop	72	0	2	61
LSD_0.05_		13	3	17

Junglerice				
Untreated control	0	100	100	100
Bispyribac-sodium	30	4	0	48
Fenoxaprop-p-ethyl + ethoxysulfuron	45	6	41	89
Penoxsulam + cyhalofop	72	16	53	89
LSD_0.05_		11	20	18

Southern crabgrass				
Untreated control	0	100	100	100
Bispyribac-sodium	30	9	82	100
Fenoxaprop-p-ethyl + ethoxysulfuron	45	0	7	65
Penoxsulam + cyhalofop	72	91	100	100
LSD_0.05_		14	15	17
